# Supplementary material for: Insulin Signaling in Intestinal Stem and Progenitor Cells as an Important Determinant of Physiological and Metabolic Traits in Drosophila
Source: Cells. 2020 Mar 26;9(4):803. doi: 10.3390/cells9040803 (PMC7226132; doi:10.3390/cells9040803)
Supplement: Supplementary file 1 [file cells-09-00803-s001.pdf]

Table S1. Statistical analysis of lifespan and stress resistance of the experimental flies with inhibited/activated IIS in ISCs and EBs.

**Esc/**		**+**	***InR-RNAi***	***Pten-RNAi***
Lifespan (days)	N	100	95	100
	Mean	26	2	24
	Difference, %		-92	-7
	χ^2		144	2.2
	p value		<0.0001	0.139
Starvation resistance (hours)	N	60	45	60
	Mean	84	47	77
	Difference, %		-60	-8
	χ^2		90	10
	p value		<0.0001	0.001
Malnutrition 1%S (days)	N	60	75	45
	Mean	7	6	6
	Difference, %		-16	-8
	χ^2		6	5
	p value		0.013	0.020
Malnutrition 1%AY (days)	N	60	45	30
	Mean	9	7	6
	Difference, %		-19	-33
	χ^2		3	11
	p value		0.081	0.001
Malnutrition 0.5%S-0.5%AY (days)	N	60	75	45
	Mean	20	9	20
	Difference, %		-56	-4
	χ^2		59	3
	p value		<0.0001	0.09

Survival between groups was compared by Log-rank test.

Table S2. Primer pairs used for qRT-PCR.

Abbreviation	Primer Pairs
rp49	F: ATCGGTTACGGATCGAACAA<br>R: GACAATCTCCTTGCGCTTCT
crq	F: CAGAGCTCTCCTCCGAATTG<br>R: ATGCCGGTGATGAGAAAGAC
dilp2	F: AGCAAGCCTTTGTCCTTCATCTC<br>R: ACACCATACTCAGCACCTCGTTG
dilp3	F: TGTGTGTATGGCTTCAACGCAATG<br>R: CACTCAACAGTCTTTCCAGCAGGG
dilp5	F: GAGGCACCTTGGGCCTATTC<br>R: CATGTGGTGAGATTCGG
dilp6	F:CCCTGGCGATGTATTTCCCAACA<br>R: CCGACTTGCACACAAATCGGTTA
akh	F: AGACCTCCAACGAAATGCTG<br>R: GTGCTTGCAGTCCAGAAAGAG
pepck	F: TCAATGGCGAATCCTGCTAC<br>R: TCCTTCACGTCCACCTTATCC
tobi	F: CCACCAAGCGAGACATTTACC<br>R: GAGCGGCGTAGTCCATCAC
bmm	F: GGTCCCTTCAGTCCCTCCTT<br>R: GCTTGTGAGCATCGTCTGGT
4ebp	F: CCAGGAAGGTTGTCATCTCG<br>R: CCAGGAGTGGTGGAGTAGAGG
upd2	F: CACAAGTGCGGTGAAGCTAA<br>R: GGCTCTT CTGCTGATCCTTG
upd3	F: GCCCTCTTCACCAAACTGAA<br>R: TTTCTTCTGGATCGCCTTTG
spi	F: CCTTCTATTTGCGCTTCGAG<br>R: CGCATGTGGTAGGGTAGCTT
krn	F: CGTGTTTGGCAACAACAAGT<br>R: TGTGGCAATGCAGTTTAAGG
vn	F: AACGCAGAGGTCACGAAGAT<br>R: GCGCACTATTAGCTCGGAAC
puc	F: CATGTGGCTAGCAATTTGA<br>R: GTCTTCGAAAAACGTACAGC
soc36	F: CAGTCAGCAATATGTTGTCG<br>R: ACTTGCAGCATCGTCGCTTC

All primers are displayed in 5′-3′ direction.

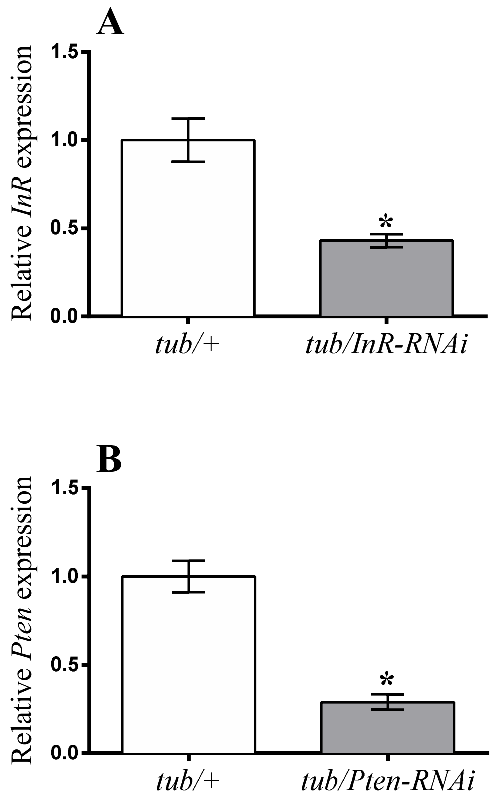

Strilbytska et al., Fig. S1

Figure S1. Relative *InR* and *Pten* expression in flies with IIS inhibition/activation in whole *Drosophila* body. The differences between means (±SEM) were compared by Student's *t*-test. Asterisk shows difference from control group (*tub*/+), $p<0.05$; n=5.

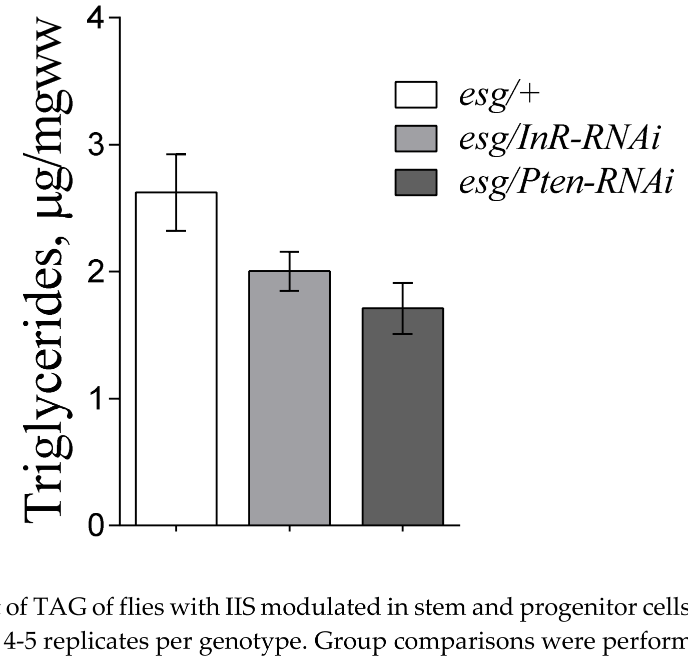

Figure S2. Amount of TAG of flies with IIS modulated in stem and progenitor cells. Results represent the mean ± SEM of 4-5 replicates per genotype. Group comparisons were performed using ANOVA followed by Newman-Keuls post-hoc test.

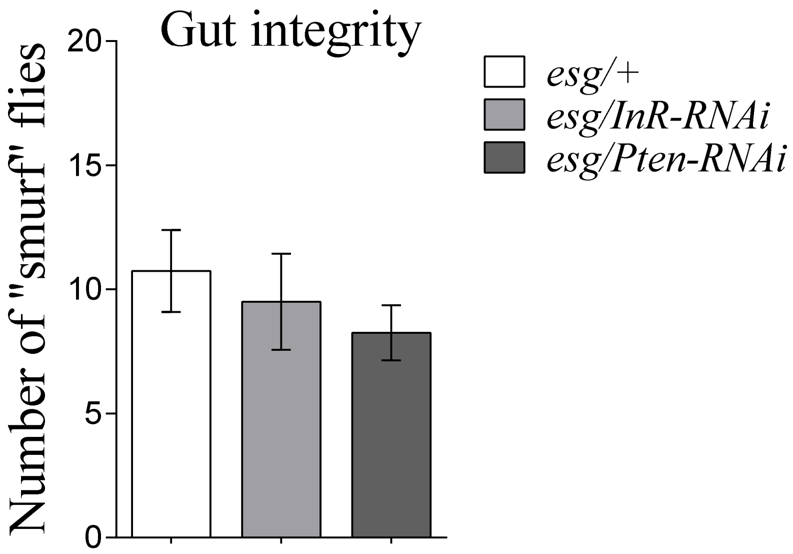

Figure S3. Number of “smurf” flies, an indicator of gut integrity, in control, *InR-RNAi*- and *Pten-RNAi*-expressing flies. The measurements were repeated every 5 days for up to 30 days (on 1, 5, 10, 15, 20, 25, 30 day). However, data for *esc*/*InR-RNAi* genotype represent the results of measurements made on the 1-st experimental day (seventh days after fly switch to 29°C and induction of RNAi expression). Bars represent mean values ± SEM for 5 replicates.
